# STED nanoscopy with fluorescent quantum dots

**DOI:** 10.1038/ncomms8127

**Published:** 2015-05-18

**Authors:** Janina Hanne, Henning J. Falk, Frederik Görlitz, Patrick Hoyer, Johann Engelhardt, Steffen J. Sahl, Stefan W. Hell

**Affiliations:** 1 German Cancer Research Center (DKFZ), Optical Nanoscopy Division, Im Neuenheimer Feld 280, Heidelberg 69120, Germany; 2 Department of NanoBiophotonics, Max Planck Institute for Biophysical Chemistry, Am Fassberg 11, Göttingen 37077, Germany

## Abstract

The widely popular class of quantum-dot molecular labels could so far not be utilized as standard fluorescent probes in STED (stimulated emission depletion) nanoscopy. This is because broad quantum-dot excitation spectra extend deeply into the spectral bands used for STED, thus compromising the transient fluorescence silencing required for attaining super-resolution. Here we report the discovery that STED nanoscopy of several red-emitting commercially available quantum dots is in fact successfully realized by the increasingly popular 775 nm STED laser light. A resolution of presently ∼50 nm is demonstrated for single quantum dots, and sub-diffraction resolution is further shown for imaging of quantum-dot-labelled vimentin filaments in fibroblasts. The high quantum-dot photostability enables repeated STED recordings with >1,000 frames. In addition, we have evidence that the tendency of quantum-dot labels to blink is largely suppressed by combined action of excitation and STED beams. Quantum-dot STED significantly expands the realm of application of STED nanoscopy, and, given the high stability of these probes, holds promise for extended time-lapse imaging.

## Abstract (web summary)

STED nanoscopy enables sub-diffraction imaging with a wide range of fluorescent probes. Here, the authors show that a bright and very photostable class of fluorescent quantum dots can be super-resolved with STED as biolabels in cellular contexts.

## Graphical abstract

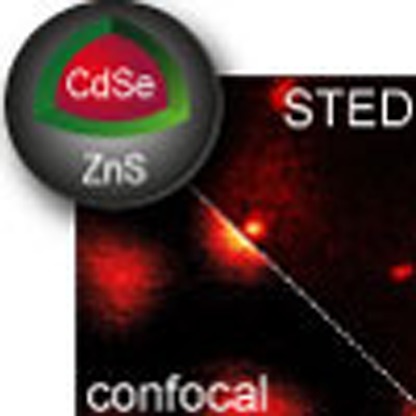

The development of STED (stimulated emission depletion) nanoscopy at the turn of this century demonstrated that far-field (i.e., lens-based) fluorescence imaging^1^ can discern features at deeply sub-diffraction (nanometre) length scales, contrary to what had been believed until then for more than a century^2^. At their most fundamental level, STED nanoscopy^3^,^4^ and other fluorescence ‘super-resolution' methods defy the diffraction resolution limit by utilizing a molecular state transition for the purpose of feature separation. Concretely, feature molecules residing closer together than half the wavelength of light are made to occupy different molecular states when they are interrogated by the same excitation diffraction pattern. In the simplest case, the two states are a fluorescent (‘on') and a non-fluorescent (‘off') state, and the feature molecules are being made to emit consecutively, enabling time-sequential readout of sub-diffraction-sized sample regions. As a result of this paradigm shift, super-resolution performance critically depends on the properties of the fluorophores in use.

STED nanoscopy requires fluorophores that can be cycled many times between their ground state S_0_ and the excited fluorescent state S_1_ which, in this concept, represent the ‘off' and the ‘on' states, respectively. While the S_1_ state is reached by optical excitation from the S_0_ state, the S_0_/S_1_ (‘off'/'on') state difference in the sample is induced by the inverse transition S_1_→S_0_, namely stimulated emission. To this end, the optical beam inducing stimulated emission, the so-called STED beam, is in most implementations doughnut-shaped, featuring a central intensity minimum (ideally, a zero of intensity) and a maximum intensity *I*, which is significantly higher than the value *I*_s_ required to make the transition S_1_→S_0_ occur with almost definiteness. Consequently, only fluorophores located within a region of diameter 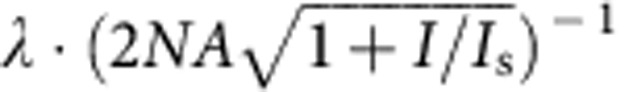
<<
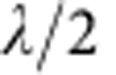
 around the central zero-intensity point of the doughnut can occupy the S_1_ state and fluoresce^2^. Here *λ* denotes the wavelength of the STED light and *NA* is the numerical aperture of the lens^5^. The wavelength for STED action needs to be tuned to an energy where excitation is negligible and stimulated emission is predominant, that is, to the red edge of the fluorophore emission spectrum^6^.

Because stimulated emission is a very fundamental molecular transition, STED nanoscopy has been demonstrated with basically all major classes of fluorophores: synthetic organic compounds, fluorescent proteins, colour centres in solids and so on, except standard semiconductor quantum dots (QDs). This is particularly unfortunate because QDs^7^,^8^ are bright emitters and can feature narrow emission ranges that are freely tunable across the whole visible spectrum. Moreover, they are widely available, can be made live-cell compatible by organic coatings and standard coupling chemistry^9^, and apart from transitions to metastable dark states (fluorescence intermittency or ‘blinking' with power-law statistics), they are exceptionally photostable. Indeed, photostability means that they can be transferred many times between their ground (‘off') and their fluorescent (‘on') state, which is particularly advantageous for STED.

Nonetheless, successful STED nanoscopic imaging of commercially available standard QDs has not been reported. A reason for this is the characteristically broad excitation spectrum that reaches deeply into the emission band, making it difficult to identify a wavelength at which optical de-excitation by stimulated emission or a similar transition prevails. While the absorbance falls off strongly towards 700 nm and beyond for the QD shown in Fig. 1, it remains non-negligible, which emerges as an issue especially when higher intensities need to be applied. Because of direct excitation by the STED beam and their characteristic bandgap structure, QDs were long thought to be unsuitable for STED nanoscopy. Therefore, not surprisingly, only two reports to date describe the application of STED-like approaches to QDs^10^,^11^. However, the QDs used in these studies were not of the type commonly used in biomedical research. They were either manganese-doped^10^ or synthesized with an exceptionally thick shell (‘giant')^11^, which seriously hampered their application.

Here we show that commercially available and widely used ZnS-coated CdSe QDs as well as CdTe QDs can be readily super-resolved by recent implementations of the STED concept. We quantify the parameters of operation leading to sub-diffraction resolution and apply this resolution to the imaging of QD-immunostained proteins in cells. We furthermore observed a strong gain in QD signal stability during STED beam action. The exceptionally high photostability of QDs even under high laser intensities in STED nanoscopy has important implications for super-resolving QD-based molecular tracking and time-lapse imaging.

## Results

### The QD fluorescent state can be counteracted optically

**Figure 1:**
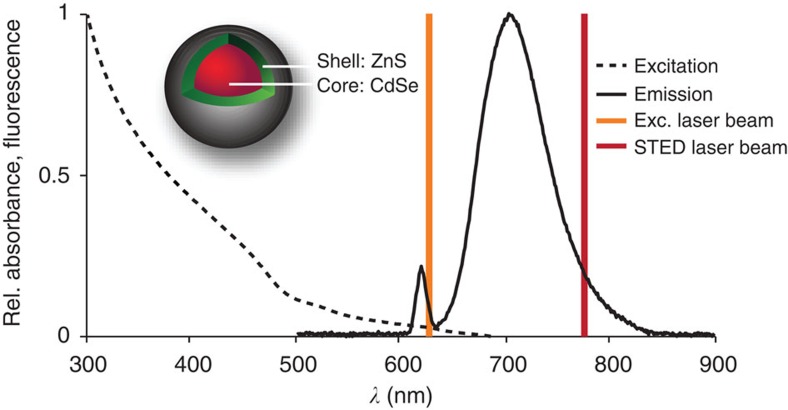
Properties of Qdot705. The excitation (Exc.; black dotted line) and the emission (black solid line) spectra of the antibody-coupled ZnS-coated CdSe Qdot705 (inset) are depicted together with the excitation laser line at *λ*_exc_=628 nm (yellow) and the STED laser line at *λ*_STED_=775 nm (red) used in this study. Rel., relative.

**Figure 2:**
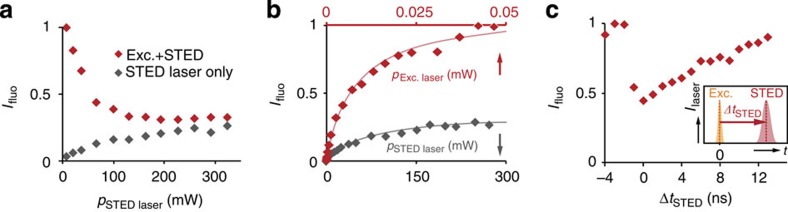
Quantification of QD fluorescence inhibition by the STED laser beam. (**a**) The fluorescence intensity (*I*_fluo_) of a dense Qdot705 sample decreases when imaged with the excitation (Exc.; *λ*_exc_=628 nm) in combination with a STED laser beam (*λ*_STED_=775 nm) at increasing STED laser power (red). The STED laser beam only also induces fluorescence of the QDs (grey). (**b**) *I*_fluo_ induced by the STED laser beam (grey) and by the excitation laser beam (red). (**c**) The STED laser pulse was translated in relation to the excitation laser pulse (inset) and *I*_fluo_ was plotted against the time delay (Δ*t*_STED_) between both laser pulses.

We found that the fluorescence ability of standard QDs (Qdot705 by Life Technologies GmbH, Darmstadt, Germany; Fig. 1) with an ∼650–770 nm emission band can be non-destructively counteracted by applying a 775-nm-pulsed laser beam of 1.2 ns pulse duration acting as the STED beam. The QDs were excited at 628 nm using a pulse train of 38 MHz and ∼1.2 ps pulse duration. The excitation and STED pulses were synchronized to ensure maximal fluorescence inhibition. While it was non-destructive and reversible, fluorescence inhibition was not complete. At >200 mW STED beam average power, a baseline of ∼30% of the original maximum fluorescence value remained (Fig. 2a). Interestingly, about the same baseline was reached when exclusively applying the STED beam, indicating that this residual fluorescence is primarily due to excitation by the STED beam itself. Nonetheless, excitation by the STED beam has a cross-section that is lower by several orders of magnitude; while the QD fluorescence signal saturated at an average power of ∼0.05 mW of 628 nm excitation light, 300 mW was necessary to saturate the QD at 775 nm (Fig. 2b). In any case, the maximal fluorescence produced at 775 nm was only 26% of that produced at 628 nm, indicating that a dynamic equilibrium is established between excitation and de-excitation, with de-excitation prevailing.

To elucidate the inhibition mechanism, we varied the temporal delay between the excitation and inhibition pulses (Fig. 2c). We found that a negative delay, that is, the inhibition pulses arriving before the excitation pulses, does not decrease the fluorescence signal. The decrease is maximal for overlapping pulses, and becomes less pronounced as the inhibition pulse is delayed with respect to its excitation counterpart, reflecting the ∼8-ns lifetime of the fluorescent state. This experiment proves that the STED beam directly inhibits the spontaneous radiative decay of the QD fluorescent state. The fact that the fluorescent state can be populated and quenched at the 38-MHz repetition rate, that is, at time intervals of the order of the fluorescent state lifetime, shows that the STED pulse effectively transfers the QDs to their ground state.

### Reversible state transfer enables sub-diffraction resolution

**Figure 3:**
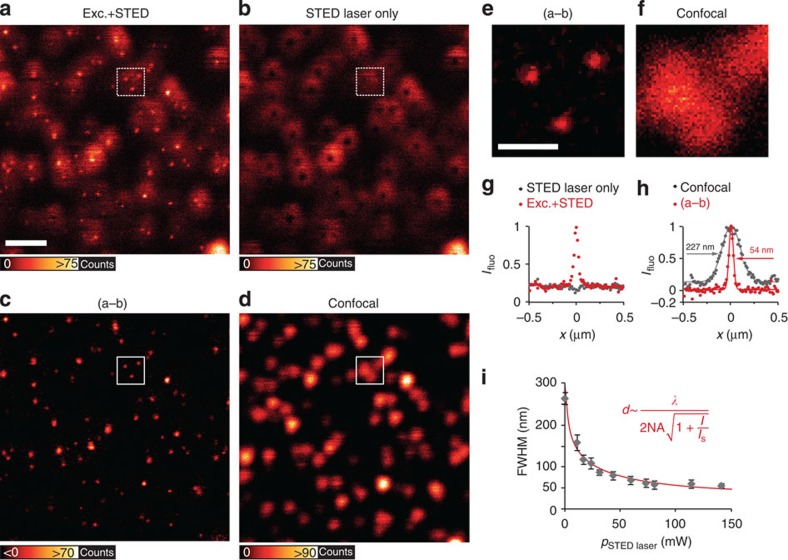
Resolution increase in STED nanoscopy of fluorescent QDs. (**a**) Qdot705 imaged in STED nanoscopy with *λ*_exc_=628 nm excitation (Exc.) and *λ*_STED_=775 nm STED wavelength. Scale bar, 1 μm. (**b**) The same sample position as in **a** imaged only with the doughnut-shaped STED laser. Images in **a** and **b** were acquired by line-wise multiplexing to avoid drift between both images. (**c**) The image acquired with illumination by the STED laser beam only (**b**) was subtracted from the STED image (**a**) to result in a background-free final image (3 × 3 median filter). (**d**) Confocal image of the same sample region for comparison. (**e**) Inset from **c**, white square, with distinct QDs that could not be separated by confocal imaging (**f**). Scale bar, 300 nm. (**g**,**h**) Averaged line profile of 10 QDs in raw images (**a**,**b**) in subtracted image (**c**) and confocal image (**d**). (**i**) The full-width at half-maximum (FWHM) of single QDs (*n*=10) as function of the STED laser power can be well approximated by the STED resolution formula shown. Error bars represent±1 s.d.

Next we used this optically controlled cycling between the ground and the excited state for imaging QDs with sub-diffraction resolution. The excitation by the STED beam (see above) is evident but minor. We therefore tested sub-diffraction imaging in a standard STED setup with *λ*_STED_=775 nm on Qdot705 with a diameter of ∼12 nm (ref. 12). We achieved resolutions ∼50 nm compared with 230 nm in confocal reference images (Fig. 3 and Supplementary Fig. 1). The super-resolved QDs are rendered as bright spots surrounded by a dim ‘halo' caused by excitation via the doughnut-shaped STED beam in raw images (Fig. 3a,g). To remove the halo and increase contrast, we imaged the same position of the sample again with only the STED laser beam turned on; this was performed in line-wise alternation to minimize drift between both images. In the image acquired with the STED laser only (Fig. 3b), each QD resembles a doughnut, with a dark centre in the middle, which is caused by scanning with the characteristic profile of the focal STED beam^13^. Subsequent subtraction of the latter yielded super-resolved background-free QD images (Fig. 3c), with the resolution on single QDs enhanced by a factor of 4.2 compared with the confocal image (Fig. 3d–f,h and Supplementary Fig. 1a). In accordance with the STED resolution formula (Fig. 3i), we found an inverse square-root dependence of achieved resolution on STED laser intensity *I*.

Besides Qdot705, other QDs with a CdTe core but no shell and emission maxima at either *λ*=700 or 720 nm, could be clearly super-resolved to ∼100 nm using the described strategy (Supplementary Fig. 2). However, QDs with emission maxima <700 nm made from either CdTe or CdSe coated with ZnS did not exhibit resolution enhancements. The overlap of the STED laser beam with the emission spectrum of the QDs therefore seems to be crucial for super-resolution, whereas composition appears to be of minor importance.

**Figure 4:**
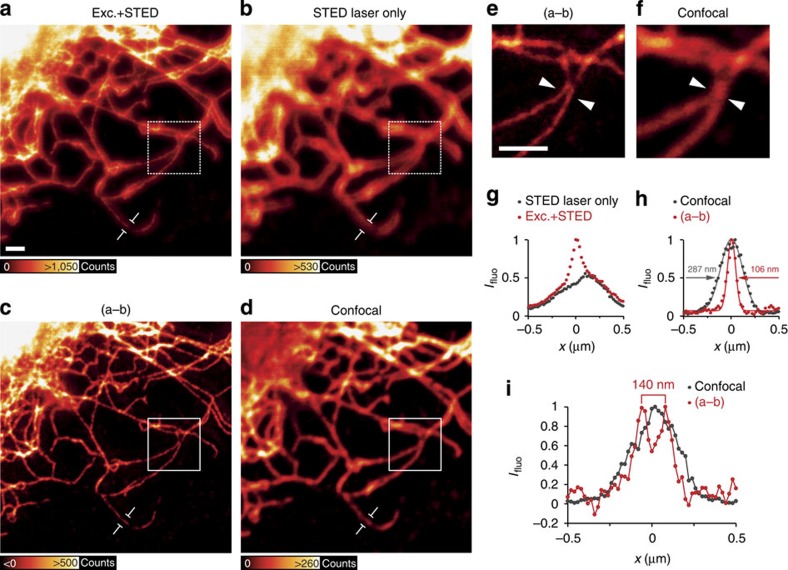
Immunofluorescence staining of cellular vimentin fibres with Qdot705. (**a**) Vimentin fibres in REF cells immunostained with Qdot705 and imaged with the excitation (Exc.) plus the STED laser beam. Scale bar, 1 μm. (**b**) QDs imaged with the STED laser beam only. Images in **a** and **b** were acquired by line-wise multiplexing. (**c**) Subtraction of the STED laser beam-only image (**b**) from the excitation plus the STED laser beam image (**a**) (3 × 3 median filter). (**d**) Confocal image of the same region. Note that the lookup tables were adjusted for better visualization. Images in **e** and **f** are insets from white squares in **c** and **d**, respectively. Scale bar, 1 μm. (**g**) Intensity profile averaged 200 nm along a single vimentin fiber indicated in **a** and **b**. (**h**) Profile as in **g** for the subtracted image (**c**) and the confocal image (**d**). (**i**) Line profile of the position indicated by arrow heads in **e** and **f**. The two fibres, resolved to a distance of 140 nm in the super-resolved image, appear as a single blurred object in the confocal reference image.

A great advantage of conventional QDs is that they are commercially available in different bio-functionalized forms to be used in a cellular context. The super-resolution performance of Qdot705 was reproducible also in immunostained cellular samples. Vimentin fibres were labelled with Qdot705 and imaged in the STED setup (Fig. 4). The resolution was significantly enhanced, yielding a 2.7-fold reduction in the measured filament diameter (Fig. 4h). If accounting for the diameter of immunostained vimentin, coated by primary and secondary antibodies, a resolution on the order of 65–75 nm can be inferred. Owing to the sub-diffraction resolution achieved, additional information could be extracted compared with the confocal reference image (Fig. 4e,f,i). Again, fluorescence caused by the STED beam was removed by subtraction of a quasi-simultaneous image taken with the STED laser only from the original STED image.

### QDs are highly photostable in optical nanoscopy

**Figure 5:**
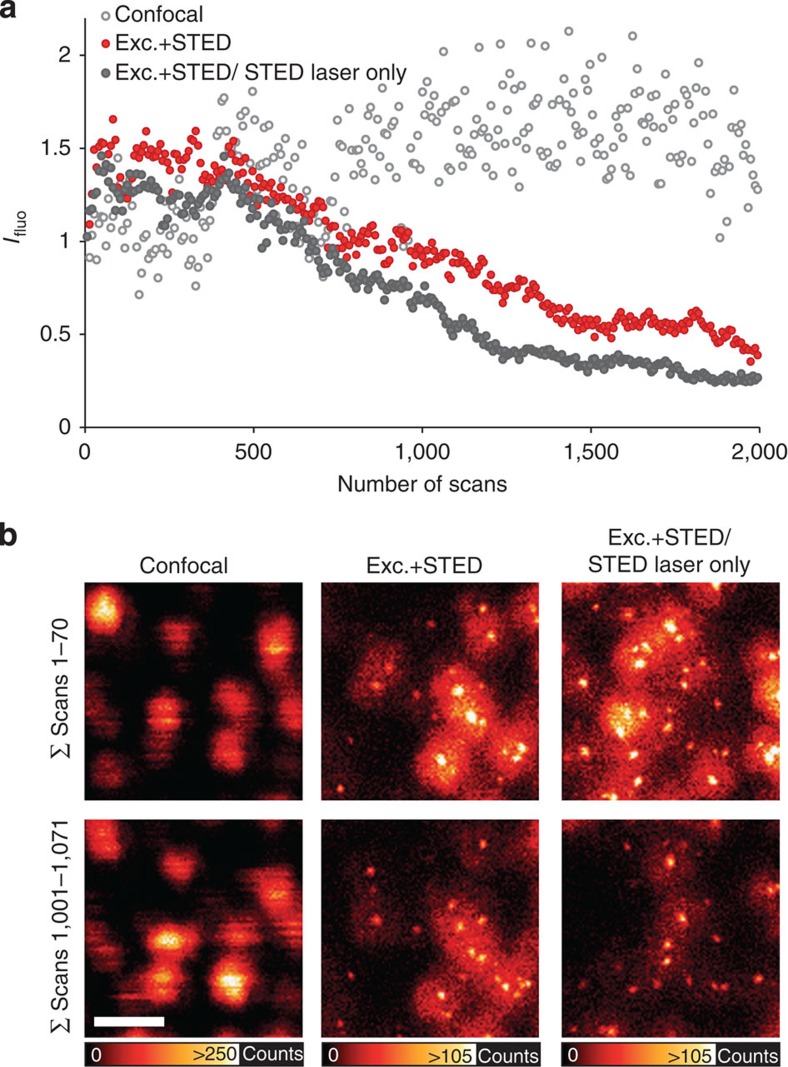
Bleaching of QDs in STED nanoscopy. (**a**) Fluorescence intensity against the number of scan repetitions for repeated scanning of QD images acquired with the excitation beam only (confocal), the excitation plus the STED laser and the excitation plus the STED laser alternating with the STED laser only, as necessary for later subtraction of the background owing to direct excitation by the STED laser. Excitation laser power was 21.8 μW, STED laser power was 344 mW and dwell time was 30 μs. (**b**) Summation of 70 scans from 1 to 70 (upper panel) and from 1,001 to 1,071 (lower panel) for all three laser settings, without subtraction of the direct excitation by the STED laser. Scale bar, 100 nm.

Owing to their remarkable photostability^14^, QDs are widely used in time-lapse or tracking experiments^15^,^16^. We investigated the bleaching resistance in our STED setup and found that Qdot705, when imaged with the excitation beam only, did not show any bleaching even after 2,000 repetitive scans of the same sample position (Fig. 5). Imaging QDs with the excitation and the STED laser beam still allowed for ∼1,300 scans before the fluorescence intensity had subsided to half of the original fluorescence intensity. Approximately 1,000 scan cycles of the imaging routine necessary for removal of fluorescence generated by the STED beam (with excitation plus STED laser, followed by another scan with the STED laser only) could be executed before fluorescence dropped to 50%. In all scanning modes, QD brightening was observed after a few scan cycles, a well-known QD property^17^. Interestingly, when excited with the excitation laser beam only, the total emission signal per frame varied strongly over time due to blinking of single QDs. However, when imaged with the additional STED laser beam, blinking appeared to be largely suppressed in the majority of trials (Supplementary Fig. 3). The blinking propensity appears to be variable due to unknown factors; it is an interesting subject for further investigation, given its potential practical relevance.

## Discussion

We showed that the fluorescence of conventional CdSe QDs can be obstructed by light. This effect was used to facilitate super-resolution microscopy down to 54 nm lateral resolution in a standard STED setup. In a cellular context (immunostained vimentin fibres), the resolution was also clearly enhanced. Intriguingly, QDs could be scanned more than 1,000 times in the STED mode with maximal resolution, which opens up new opportunities for applications of STED nanoscopy in time-lapse imaging or other areas, where repetitive imaging of the same sample position is needed. The effect of efficient fluorescence quenching by light was observed in several different kinds of QDs. Therefore, it should be possible to image multiple colours within one sample by either spectral or lifetime separation upon further development.

Even though the STED laser line at *λ*_STED_=775 nm is well separated from the normal excitation spectrum of Qdot705 (Fig. 1), not only fluorescence silencing but also, to a much lesser extent, excitation at this wavelength was evident in all our measurements. All evidence points to the fact that the observed signal is fluorescence induced by direct excitation with the STED beam, which manifests as doughnut-shaped halos in the periphery of QDs. Concretely, the signal in this halo displayed a similar lifetime as signal from the super-resolved centre (Supplementary Fig. 4). In addition, with increasing STED laser power, the signal saturated, arguing against reflection or scattering, which would be rejected by our filtering in any case (see ‘Optical setup' in Methods section). Since the power of the focal STED beam is on the order of 100 mW, two potential mechanisms could account for direct excitation with the STED laser beam: (i) multiphoton absorption or (ii) a contribution of thermal energy at room temperature which, together with the energy of the exciting 775 nm photon, makes direct excitation possible. Our results suggest that both fluorescence excitation and fluorescence silencing of the QD are induced by the 775-nm STED beam. Both processes are in competition, the silencing transition clearly being the dominant mechanism.

By direct excitation with the STED beam, the imaging contrast was impaired to some extent. Nevertheless, we found that simple subtraction of recordings of ‘fluorescence by the STED beam only' was sufficient to obtain high-resolution images at very good contrast even in densely labelled cellular samples. Note that subtraction is not always a viable option, but here it is greatly facilitated by the high photostability of the QDs.

Whether the underlying photophysical mechanism for sub-diffraction resolution enhancement in the case of QDs is in fact stimulated emission was not proven beyond a doubt in this first study. However, our results clearly point towards this mechanism. In particular, we found that the STED laser beam predominantly acts on excited fluorophores. The existence of additional energy levels within the QD as proposed in ref. 10 could not be excluded. For quasi-overlapping pulse pairs, time gating of fluorescence^18^ had no strong effect on the resolution as demonstrated by apparent diameters (full-width at half-maximum) of individual QD images inferred from Gaussian fitting (Supplementary Fig. 1b). Mean STED resolution was however slightly improved for short gate delays of ∼1–3 ns avoiding detection during the STED pulse. For longer gate delays, too close to the (average) QD fluorescent lifetime, the reduced signal compromised effective resolution and no gating gain could be demonstrated. While stimulated emission is the most probable cause for the fluorescence inhibition, we note that the actual state transition is rather irrelevant for gaining super-resolution. As long as the occupation of the fluorescent state is reversibly and non-destructively prevented by the doughnut beam, the concept is viable. The inverse dependence of the resolution on the square-root of the STED laser intensity proves that the gain in resolution is driven by the doughnut-shaped STED beam.

Especially in the context of long-term image acquisition and particle tracking, QD blinking represents a major limitation, and numerous studies describe different approaches to reducing blinking either by chemical additives to the imaging medium or by extra coatings to shield the semiconductor core from the environment^19^,^20^. Figure 5a and Supplementary Fig. 3 document our observation that blinking was strikingly reduced under combined illumination with the excitation laser and a long-wavelength laser at 775 nm; however, we faced difficulties in reproducing this finding in a controlled manner, possibly due to QD ageing or slight variations in sample mounting conditions. If indeed there is a causality between illumination pattern and QD fluorescence intermittency, this could be the first light-based method to suppress blinking, adding to the appeal of QD imaging by STED. Finally, showing that QDs can be used in STED nanoscopy underscores the universality of this super-resolution concept beyond classical organic fluorophores and fluorescent proteins, thus further expanding its realm of application, with numerous benefits from biomedicine to materials research.

## Methods

### Quantum dots

The QDs used in this study were as follows: Qdot 655 goat whole IgG anti-chicken IgY (H+L) conjugate, Qdot705 goat F(ab′)2 anti-rabbit IgG conjugate (H+L; both obtained from Life Technologies (Carlsbad, CA, USA)) and CdTe QDs 680 nm (hydrophilic), CdTe QDs 700 nm (hydrophilic), CdTe QDs 720 nm (hydrophilic; all obtained from PlasmaChem GmbH (Berlin, Germany)).

### Sample preparation

QDs diluted in ddH_2_O were incubated on coverslips coated with poly-L-lysine for 10 min. After washing with ddH_2_O, samples were mounted in Mowiol/Dabco as described in ref. 21.

For vimentin staining, rat embryonic fibroblasts were fixed in ice-cold methanol for 15 min. Blocking was performed with 3% bovine serum albumin and 0.3% Triton-X in PBS for 30 min. Fixed cells were then incubated consecutively with primary rabbit anti-vimentin monoclonal antibody (Abcam, Cambridge, UK) and with secondary Qdot705 goat F(ab′)2 anti-rabbit IgG, both diluted 1:50 in blocking solution for 1 h each, and mounted in Mowiol/Dabco.

### Optical setup

All experiments were performed on a custom-built STED setup. For excitation, a super-continuum laser with 38.6 MHz pulse rate was used. The operating wavelength at *λ*_exc_=628 nm and its intensity was chosen by an acousto-optical tunable filter (AOTF) and refined by a clean-up filter (610/49). Other excitation wavelengths were tested but were found not to influence imaging results. The custom-built STED laser with *λ*_STED_=775 nm wavelength (MPB Communications Inc., Montreal, Canada), a pulse length of 1.2 ns and 1,200 mW maximal output power was co-aligned with the excitation laser via a 775-nm short-pass filter. Both laser beams passed a polarization-maintaining optical fiber and were focused into a QuadScanner developed in-house^22^. The beams were led through an easySTED phase plate (630/775 nm, B-Halle, Berlin, Germany) in the back focal plane of the 100 × objective (HCX PL Apo100 × /1.44 OIL Corr CS, Leica Microsystems). The back-propagating sample fluorescence was collected by the same objective and led into the detection path by a directional beam splitter^23^ and refined by a pinhole. Light between *λ*=680 and 740 nm was collected on a single-photon-counting module (SPCM-AQRH, PerkinElmer, Rodgau, Germany). To block the STED laser wavelength, two 775 nm short-pass filters were positioned after the pinhole. The optical system was controlled via an field-programmable gate array (FPGA) board and in-house software written in LabVIEW 10.0 (National Instruments, Austin, Texas, USA).

### Image acquisition and analysis

Images were acquired with time-gated detection, with the gate opening after the excitation and the STED laser pulses have passed. Acquisition of images was usually accomplished with multiple line and frame accumulation. A typical acquisition setting would be accumulation of seven lines and three frames in total, with a pixel dwell time of 30 μs. Each image was scanned with the (i) excitation plus the STED laser beams, and (ii) the STED laser beam only in line-wise multiplexing. Resulting images were subsequently subtracted to correct for fluorescence elicited by the STED laser. All values for laser power given refer to the power in the focal plane as estimated by measuring the power before entering the microscope stand and assuming 36% loss on the way through the optics. To compare fluorescence intensities, a confocal image (excitation laser only) at low laser powers was taken first to which further images were normalized to correct for variable sample brightness. For all experiments except the bleaching and resolution measurements, a new spot was chosen for every image to avoid bleaching artefacts. To estimate fluorescence lifetimes, fluorescence was collected in two subsequent time gates as described in ref. 23.

## Additional information

**How to cite this article:** Hanne, J. *et al.* STED nanoscopy with fluorescent quantum dots. *Nat. Commun.* 6:7127 doi: 10.1038/ncomms8127 (2015).

## Supplementary Material

Supplementary Information Supplementary Figures 1-4
