# Supplementary material for: STED nanoscopy with fluorescent quantum dots
Source: Nat Commun. 2015 May 18;6:7127. doi: 10.1038/ncomms8127 (PMC4479004; doi:10.1038/ncomms8127)
Supplement: Supplementary Information — Supplementary Figures 1-4 [file ncomms8127-s1.pdf]

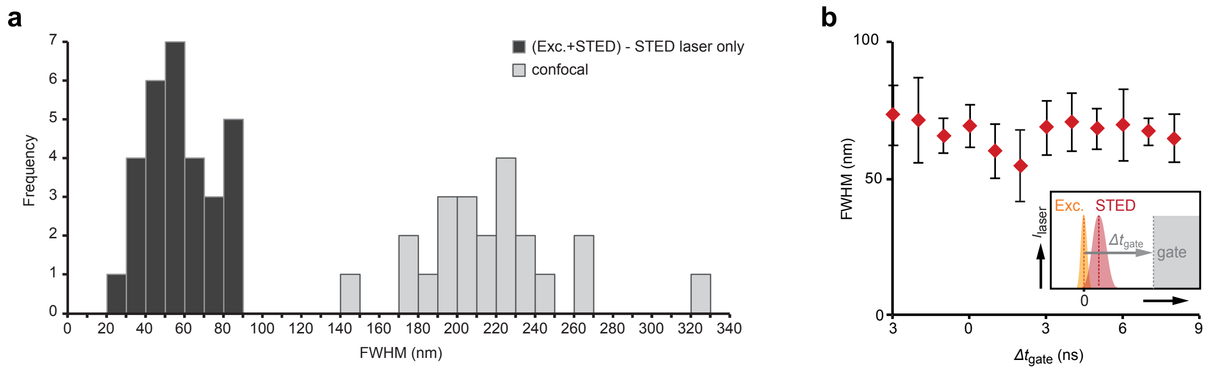

Supplementary Figure 1 | Resolution enhancement: inferred size of single QD images. (**a**) FWHMs of individual QDs from Fig. 3 were measured and plotted in a histogram for the subtracted STED image (dark gray) and the confocal image (light gray; bin size 10 nm). **(b)** FWHM from QDs (n=10 determinations each, error bars represent ± one standard deviation.) for variable time delay ("gating delay") before the onset of photon gating for 10 ns.

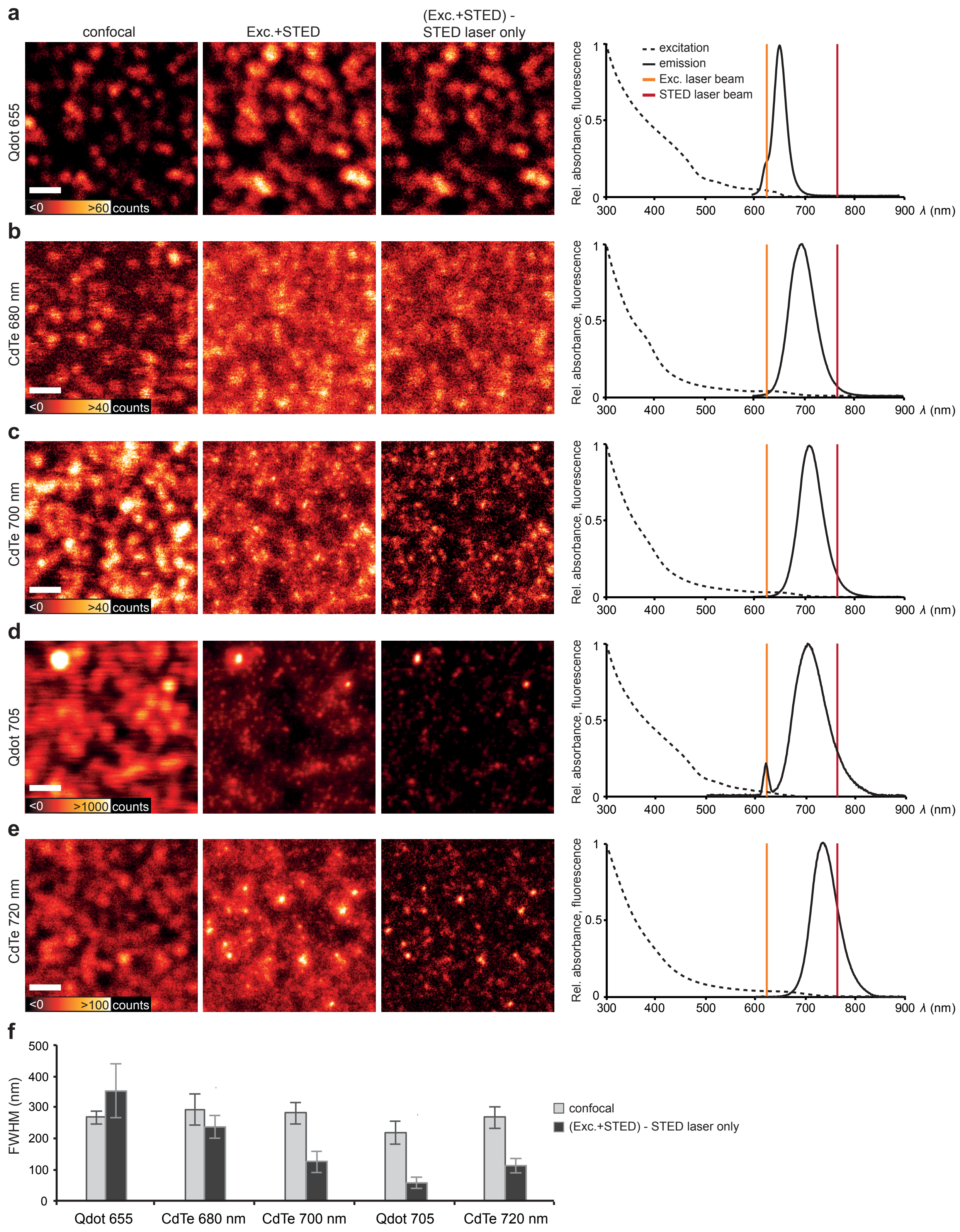

Supplementary Figure 2 | STED nanoscopy with different types of fluorescent QDs.

Supplementary Figure 2 (description) |

STED nanoscopy with different types of fluorescent QDs.

Confocal, STED and subtracted image as well as excitation (dotted line) and emission (solid line) spectra, the excitation (yellow) and STED (red) wavelength of **(a)** Qdot655, **(b)** CdTe quantum dot 680 nm, **(c)** CdTe quantum dot 700 nm, **(d)** Qdot705 and **(e)** CdTe quantum dot 720 nm are shown. While in (a) and (b) no resolution enhancement could be detected in the STED image, (c-e) exhibit clearly improved resolution to different extents. (f) FWHM of Qdots in a-e were determined for each QD type (*n*≥10, error bars represent ± one standard deviation). Scale bars: 1 µm.

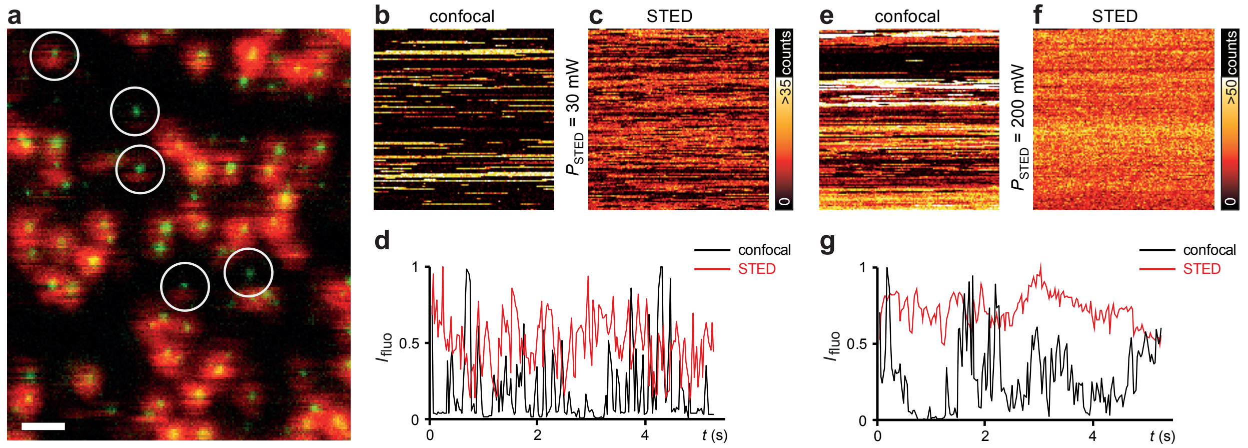

Supplementary Figure 3 | Fluorescence blinking suppression in STED nanoscopy. **(a)** Overlaid confocal (red) and STED (green) image. Dark horizontal lines in the image result from fluorescence intensity fluctuations (signal intermittency) of the QDs. As visible for the examples of circled QD, fluorescence off-times appear to be suppressed in the STED image. Scale bar: 600 nm. Time traces of single QDs with the scanner set in the confocal **(b,e)** and STED **(c,f)** modes recorded at two laser powers. Each pixel integrates 300 µs and the images are written from top left (earliest time point) line-wise to bottom right (latest time point). **(d,g)** Intensity line profile vs. time from the above images.

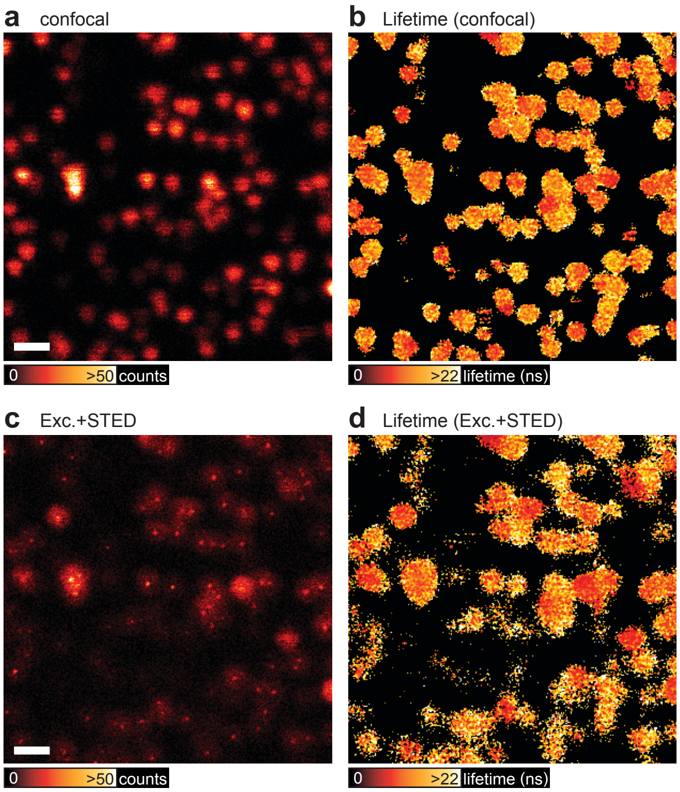

Supplementary Figure 4 | Lifetime map of confocal compared to Exc.+STED recording. Lifetimes for QDs imaged in confocal **(a)** and Exc.+STED **(c)** mode were approximated using two successive detection time gates (**b,d**; gating delay: 3 ns after excitation pulse, length of first gate: 8 ns, length of second gate: 10 ns). Similar lifetimes were determined for confocal and super-resolution images and, importantly, no difference in lifetimes is seen between the super-resolved center and the halo caused by direct STED-laser excitation in **(d)**. Scale bars: 1 µm.
